# Special Issue: “Novel Researches and Perspectives on Prostate Cancer”

**DOI:** 10.3390/ijms25042054

**Published:** 2024-02-08

**Authors:** Giovanni Luca Beretta

**Affiliations:** Department of Experimental Oncology, Fondazione IRCCS Istituto Nazionale dei Tumori, 20133 Milan, Italy; giovanni.beretta@istitutotumori.mi.it

Prostate cancer (PCa) represents the second most diagnosed tumor and the fifth most common cause of cancer death in men globally [1]. This disease ranges from indolent to aggressive tumors that may rapidly progress and metastasize to castration-resistant PCa (CRPC). Despite the effectiveness of novel pharmacological approaches, including cabazitaxel and docetaxel, abiraterone, enzalutamide, radium-223-based bone-targeting radiotherapy, ^177^Lu-PSMA-617, olaparib and sipuleucel-T, CRPC still remains incurable [2,3]. Therefore, the elucidation of the mechanisms underlying PCa development and progression and drug resistance, as well as the optimization of innovative medical interventions are urgent.

In this Special Issue, particular emphasis is placed on Prostate-Specific Membrane Antigen (PSMA) and galectin-3 as selective PCa targets for use in diagnostic or therapeutic interventions and on drug combination and immunotherapy strategies for overcoming chemotherapy resistance [4,5,6,7,8,9,10].

The article by Ahmadi et al. [4] deals with the topic of metabolic dysregulation in PCa. Elevated levels of ATP-citrate lyase (ACLY), Glucose Transporter 1 (GLUT1) and PSMA are often reported in PCa patients. The novelty of the study is the finding that, in addition to high levels of GLUT1 and ACLY, the over-expression of PSMA can also predict a shorter time to progression in patients undergoing active surveillance. This behavior is in keeping with the observation that the increased enzymatic activity of PSMA in PCa patients represents a selective growth advantage which supports rapid disease progression [11]. 

Three of the articles within this Special Issue embrace the selective targeting of PCa for theranostics [5,6,7]. The study by Lahnif et al. describes MMAE.VC.SA.617, a novel small molecule–drug conjugate for the selective targeting of PSMA-positive PCa [5]. The compound conjugates the potent antimitotic drug monomethyl auristatin E (MMAE) and the high-affinity PSMA inhibitor derivative KuE-617 via a valine–citrulline linker which is sensitive to cathepsin B’s enzymatic activity. Following cathepsin B cleavage, a lysosomal protease which is over expressed in PCa, MMAE.VC.SA.617 releases MMAE, which exerts its antitumor activity by interacting with microtubules. Although the in vivo data show translational potential, the authors underline that the chemical structure of the compound requires further optimization to ameliorate its PSMA binding affinity as well as its antitumor potency, including the introduction of a PSMA-targeting moiety with a higher affinity than KuE-617 or the introduction of linkers which can enhance its lipophilicity and, in turn, membrane diffusion. Along the same vein, Hasnowo and co-workers describe the synthesis of a novel urea-based PSMA inhibitor (N-[N-[(S)-1,3-dicarboxypropyl]carbamoyl]-(S)-L-lysine) conjugated via a linker containing two phenylalanine residues with (tributylstannyl)benzoic acid groups for iodine-123 radiolabeling [6]. Following in vivo administration, the conjugate seeks out PSMA-expressing cells. Compared to ^177^Lu-PSMA-617, which has been shown to be very promising in clinical trials [12], the new radioligand demonstrates a more favorable bio-distribution, suggesting a potential use within PCa diagnosis. The radioligand is very promising for image-guided surgery on PSMA-positive PCa lesions. Similarly, the article by Cacaccio et al. deals with drawbacks that are typical of photofrin-photodynamic therapy (PDT), including the scarce PCa specificity and the depth light penetration at 630 nm, and proposes the conjugation of 3-10(hexyloxy)ethyl-3-devinylpyropheophorbide-a (HPPH) with a galactose residue for the targeting of galectin-3, which is expressed in PCa [7,13]. In addition to its role in galectin-3-mediated PCa targeting, the conjugate acts either as a photo sensitizer for PDT in the near infrared irradiation or as a sono sensitizer for sonodynamic therapy (SDT). In contrast to PDT, SDT is oxygen independent, and this feature is interesting for the treatment of hypoxic tumors. 

An overview of the emerging immunotherapy approaches is given by Meng and co-workers [8]. In contrast to highly immunogenic malignancies, the immunosuppressive properties characterizing the microenvironment of PCa promote immune evasion, and this peculiarity has delayed the use of immunotherapy in the treatment of this disease [14]. After decades of limited therapeutic efficacy, encouraging clinical outcomes have been reached following the introduction of checkpoint inhibitors into clinical practice, including bispecific antibodies, CAR T cell therapy and therapeutic vaccines, as well as cytokines. Despite this success, immunotherapy interventions have yet to improve the survival outcomes of men suffering from PCa. Important challenges, including the immunosuppressive effects of androgen signaling and low tumor mutation burden, as well as the redundancy of many immunosuppressive mechanisms, have hampered single-agent efficacy. The review summarizes the immunotherapeutic strategies that are being used in clinical trials which are currently enrolling both CRPC and early stage PCa patients.

The acquired chemoresistance limits the therapeutic outcomes in both veterinary and human oncology, and this issue is tackled in two of the articles in this Special Issue [9,10]. An understanding of the metabolic changes underlying resistance to 2-hydroxy-flutamide in PCa is critical for creating drug combination strategies that can improve patient outcomes. The research by Mora-Rodríguez and colleagues shows how the long-term exposure of androgen-sensitive PCa cells to 2-hydroxy-flutamide stimulates the emergence of a stem cell-like quiescent features that favors cell survival in reduced phosphocholine metabolism conditions and cell-cycle arrest, in turn promoting drug resistance. Currently, there are no satisfying treatment strategies for canine prostate cancer. Therefore, Packeiser and co-workers have generated doxorubicin (DOXO)-resistant canine prostate cancer cell sublines exposing DOXO-sensitive parental cells to increased concentrations of DOXO. The mechanism driving the development of DOXO resistance in all the generated sublines is the DOXO cellular efflux mediated by the over-expression of the membrane transporter Multidrug Resistance Protein 1 (MDR1). This finding is corroborated by the observation that tariquidar, an MDR1 inhibitor, reverses the acquired DOXO-resistance. Moreover, since tyrosine kinase inhibitors potentially reverse MDR1-dependent drug-resistance, and they themselves show antiproliferative activity, a potential synergistic interaction between tyrosine kinase inhibitors and DOXO is proposed by the authors.

## References

[1] Sung H., Ferlay J., Siegel R.L., Laversanne M., Soerjomataram I., Jemal A., Bray F. (2021). Global Cancer Statistics 2020: GLOBOCAN Estimates of Incidence and Mortality Worldwide for 36 Cancers in 185 Countries. CA Cancer J. Clin..

[2] Cai M., Song X.L., Li X.A., Chen M., Guo J., Yang D.H., Chen Z., Zhao S.C. (2023). Current therapy and drug resistance in metastatic castration-resistant prostate cancer. Drug Resist. Updat..

[3] Hatano K., Nonomura N. (2023). Systemic Therapies for Metastatic Castration-Resistant Prostate Cancer: An Updated Review. World J. Mens. Health.

[4] Ahmadi E., Wang S., Gouran-Savadkoohi M., Douvi G., Isfahanian N., Tsakiridis N., Faught B.E., Cutz J.C., Sur M., Chawla S. (2023). Prostate-Specific Membrane Antigen (PSMA) Expression Predicts Need for Early Treatment in Prostate Cancer Patients Managed with Active Surveillance. Int. J. Mol. Sci..

[5] Lahnif H., Grus T., Salvanou E.A., Deligianni E., Stellas D., Bouziotis P., Rösch F. (2023). Old Drug, New Delivery Strategy: MMAE Repackaged. Int. J. Mol. Sci..

[6] Hasnowo L.A., Larkina M.S., Plotnikov E., Bodenko V., Yuldasheva F., Stasyuk E., Petrov S.A., Zyk N.Y., Machulkin A.E., Vorozhtsov N.I. (2023). Synthesis, 123I-Radiolabeling Optimization, and Initial Preclinical Evaluation of Novel Urea-Based PSMA Inhibitors with a Tributylstannyl Prosthetic Group in Their Structures. Int. J. Mol. Sci..

[7] Cacaccio J., Durrani F.A., Kumar I., Dukh M., Camacho S., Fayazi Z., Sumlin A., Kauffman E., Guru K., Pandey R.K. (2023). Excitation of a Single Compound by Light and Ultrasound Enhanced the Long-Term Cure of Mice Bearing Prostate Tumors. Int. J. Mol. Sci..

[8] Meng L., Yang Y., Mortazavi A., Zhang J. (2023). Emerging Immunotherapy Approaches for Treating Prostate Cancer. Int. J. Mol. Sci..

[9] Mora-Rodríguez J.M., Sánchez B.G., Sebastián-Martín A., Díaz-Yuste A., Sánchez-Chapado M., Palacín A.M., Sánchez-Rodríguez C., Bort A., Díaz-Laviada I. (2023). Resistance to 2-Hydroxy-Flutamide in Prostate Cancer Cells Is Associated with the Downregulation of Phosphatidylcholine Biosynthesis and Epigenetic Modifications. Int. J. Mol. Sci..

[10] Packeiser E.M., Engels L., Nolte I., Goericke-Pesch S., Murua Escobar H. (2023). MDR1 Inhibition Reverses Doxorubicin-Resistance in Six Doxorubicin-Resistant Canine Prostate and Bladder Cancer Cell Lines. Int. J. Mol. Sci..

[11] Yao V., Parwani A., Maier C., Heston W.D., Bacich D.J. (2008). Moderate expression of prostate-specific membrane antigen, a tissue differentiation antigen and folate hydrolase, facilitates prostate carcinogenesis. Cancer Res..

[12] Aggarwal R., Starzinski S., de Kouchkovsky I., Koshkin V., Bose R., Chou J., Desai A., Kwon D., Kaushal S., Trihy L. (2023). Single-dose 177Lu-PSMA-617 followed by maintenance pembrolizumab in patients with metastatic castration-resistant prostate cancer: An open-label, dose-expansion, phase 1 trial. Lancet Oncol..

[13] Gao J., Li T., Mo Z., Hu Y., Yi Q., He R., Zhu X., Zhou X., She S., Chen Y. (2018). Overexpression of the galectin-3 during tumor progression in prostate cancer and its clinical implications. Int. J. Clin. Exp. Pathol..

[14] Kwon J.T.W., Bryant R.J., Parkes E.E. (2021). The tumor microenvironment and immune responses in prostate cancer patients. Endocr. Relat. Cancer.

